# Correlation between apoptosis and TGF-β1 expression in the mucosal epithelium of rat small intestine in a cold stress state

**DOI:** 10.3892/etm.2013.983

**Published:** 2013-02-28

**Authors:** YONGJUN LI, DEAN TIAN

**Affiliations:** 1 Department of Gastroenterology, The First Affiliated Hospital of Shihezi University, Shihezi, Xinjiang 832006;; 2 Department of Gastroenterology, Tongji Hospital, Huazhong University of Science and Technology, Wuhan, Hubei 430030, P.R. China

**Keywords:** cold-restraint stress, transforming growth factor-β1, apoptosis

## Abstract

The aim of this study was to investigate the correlation between the expression of transforming growth factor-β1 (TGF-β1) in the mucosal tissue of rat small intestine and the apoptosis of epithelial cells in the small intestine in a cold-restraint stress state. Immunohistochemistry was used to detect the expression of TGF-β1. Terminal deoxynucleotidyl transferase deoxyuridine triphosphate (dUTP) nick end labeling (TUNEL) and DNA agarose gel electrophoresis were used to detect apoptosis. After 8 and 12 h of cold-restraint stress, the positive expression rate of TGF-β1 in the rat small intestine epithelial tissue was 59.09 and 54.16%, respectively. The apoptotic index (AI) of the rat small intestine epithelial cells was 25.69±8.09 and 19.65±6.61%, respectively. The positive expression rate of TGF-β1 in the epithelial tissue of the rat small intestine was positively correlated with the AI of the epithelial cells (r=0.980, P<0.05). The epithelial cells of the rat small intestine exhibited apoptosis under cold-restraint stress. TGF-β1 is one of the key factors that induces apoptosis of the epithelial cells of the rat small intestine.

## Introduction

A complex signaling network composed of multiple signaling molecules and pathways extensively and accurately regulates the balance between cell proliferation, differentiation and apoptosis (1), playing a critical role in the normal development of cells and bions (2). When this balance is broken, abnormal cell apoptosis and growth, as well as pathological events occur. Therefore, investigating the basic mechanisms responsible for maintaining and destroying the balance between cell proliferation, differentiation and apoptosis is significant for understanding the processes of various physiological and pathological events. Transforming growth factor-β (TGF-β) and its mediated pathways are important parts of the signaling network that regulates the physiological functions of bions and cells (3).

TGF-β belongs to a cytokine superfamily that regulates cell proliferation, differentiation and apoptosis (4). The activity center of TGF-β is a 25-kDa dimer structure composed of two identical 12.5 kDa peptide chains, linked by a disulfide bridge. At least five subtypes of TGF-β, including TGF-β1, 2, 3, 4 and 5, have been identified in mammals. The majority of cells express at least one type of TGF-β (5). Therefore, TGF-β is a multifunctional factor widely expressed *in vivo*. One study demonstrated that TGF-β is widely involved in the pathophysiological process and exerts significant effects on cell proliferation and differentiation *in vivo*, production of extracellular matrix, wound healing, embryonic development, angiogenesis, fibrosis, apoptosis and the immune system (6). TGF-β1 has a major presence in the human body and inhibition of the TGF-β1 signaling pathway may occur as a result of mutation. Moreover, inactivation of TGF-β1 and its receptor gene is one of the main causes of cancer development.

The TGF-β1 receptor is a transmembrane glycoprotein present on the cell surface and is widely distributed on the surface of normal tissues and tumor cells. TGF-β1 has five receptors with unique structures (types I, II, III, IV and V), among which types IV and V are identified only in pituitary cells and bovine liver. The first three receptors are currently widely studied. Receptor types I and II (TRβ I and TRβ II) are transmembrane serine/threonine kinases that initiate intracellular signaling via phosphorylation of Smads. Receptor types I and II are composed of an extracellular ligand-binding domain and a domain with serine/threonine kinase activity involved in intracellular signal transduction. Receptor type III is not directly involved in the process of signal transfer. TGF-β1 exerts an effect by binding to TGF-β1 receptors. Studies have shown that the dimers formed by receptor types I and II have a leading role in the TGF-β1 signaling pathway (4). TGF-β1 is a pleiotropic polypeptide that positively regulates biological processes and negatively regulates cell proliferation, differentiation and apoptosis. The biological effects of TGF-β1 are dependent on cell type and physiological conditions. The absence of TGF-β1 signaling in T cells causes spontaneous differentiation of T cells and autoimmune disease (7), suggesting that TGF-β1 signaling is required for maintaining the inner balance of T cells. TGF-β1 strongly inhibits the proliferation of epithelial cells. TGF-β1 has also been reported to induce apoptosis in certain cells, including liver and liver tumor cells (8). By contrast, TGF-β1 has anti-apoptotic effects and is able to elevate the survival rate of cells in certain cases. Research on the TGF-β family and their mechanism of action has always gained significant interest due to the importance, diversity and uncertainty of the role of TGF-β1.

The mechanism of TGF-β1-mediated signal transduction via interaction with receptors remains unclear. Smad-mediated signaling pathways are considered the main signaling pathways by which TGF-β1 produces its biological effects (9). Previously, a study identified that extracellular signal-regulated kinases (ERKs), c-Jun N-terminal kinases (JNKs) and p38 mitogen-activated protein kinases (MAPKs) are involved in TGF-β-mediated signal transduction events (10) and have significant roles in a variety of the biological effects mediated by TGF-β.

Apoptosis is a basic physiological mechanism for maintaining homeostasis in an organism. The body eliminates damaged, aged and mutant cells by apoptosis to maintain physiological balance. An abnormal apoptosis level in tissues (too high or too low) leads to the occurrence of various diseases. The close correlation between apoptosis and disease has received focus in the medical field. In addition, apoptosis and its mechanism have been widely investigated, including the effect of TGF-β1 on the mechanism of apoptosis. In T cells, TGF-β1 inhibits Fas ligand-induced apoptosis (11). Domestic studies concerning the effect of TGF-β1 on the apoptosis of epithelial cells of the small intestine are few. Thus, the current study was designed to detect the expression of TGF-β1 in the epithelial tissue of the rat small intestine in a cold-restraint stress state, as well as the apoptosis of the epithelial cells to clarify the effect of TGF-β1 on the apoptosis of the rat small intestine epithelial cells and provide a theoretical basis for an improved understanding of the role of TGF-β1 in apoptosis.

## Materials and methods

### Animals

A total of 60 male Sprague-Dawley rats weighing 180–220 g were randomized into three groups: the normal control (NC), cold-restraint stress (CS) and CS drug intervention (DCS) groups. The rat model of cold-restraint stress was established as follows: after fixation of limbs on a bracket, the rat was immersed in cold water (constant temperature, 18°C), with sternum parallel to the water surface. After 2 h of cold-restraint stress, the rat was taken from the water and the restraint was removed. The rat was then placed in a dry and warm cage until the normal state was recovered. The CS and DCS groups were further randomized into five subgroups according to the time points (2, 4, 8, 12 and 24 h) after the establishment of a successful model. From 1 week before the establishment of the CS model, the DCS group received a single dose of 20% decorin (Shanghai Deli Biotechnology Co., Ltd., Shanghai, China) at 0.5 ml/kg into the ranine vein at intervals of 12 h for 7 days, to specifically inhibit the activation of TGF-β1. The CS models were then established for the CS and DCS groups. The experiments were approved by Animal Ethics Committee of the First Affiliated Hospital of Shihezi University (Shihezi, China).

### Methods

The apoptotic genome in the ileal mucosa epithelial cells was extracted using a rapid animal genomic DNA extraction kit (Beijing extensive biological Tektronix Gene Technology Co., Ltd., Beijing, China) and then subjected to DNA agarose gel electrophoresis. Apoptosis of the cells was detected using terminal deoxynucleotidyl transferase deoxyuridine triphosphate (dUTP) nick end labeling (TUNEL; kits were purchased from Roche Company, Basel, Switzerland). The terminal segment of the ileal mucosal tissue was embedded with paraffin and then sectioned. TGF-β1 was observed using the immunohistochemistry streptavidin-peroxidase method (kits were provided by Wuhan Boster Biological Engineering Co., Ltd., Wuhan, China).

### Statistical analysis

SPSS 10.0 was used for statistical analysis. The χ^2^ test was used for counting data. The t-test and analysis of variance were used for measurement data. Spearman correlation analysis was used for the correlation analysis. P<0.05 and P<0.01 were considered to indicate a statistically significant and highly statistically significant result, respectively.

## Results

### Agarose gel electrophoresis of genomic DNA

The bands of the DNA genome in the NC group were detected near the wells. Characteristic ladder bands were detected in the CS group using agarose gel electrophoresis at 8 and 12 h after modeling. For the DCS group, the characteristic ladder bands were not detected using agarose gel electrophoresis at each time point.

### Immunohistochemical staining results

The positive expression rates of TGF-β1 in the epithelial tissue of the ileal mucous membrane at two of the time points, 8 and 12 h, for the CS group, were significantly different to that of the NC group (P<0.05; Table I).

No significant difference was observed in the positive expression rate of TGF-β1 in the epithelial tissue of the ileal mucous membrane at each time point in the DCS group compared with that in the NC group (P>0.05; Table II).

### Detection of apoptosis

**Figure 1:**
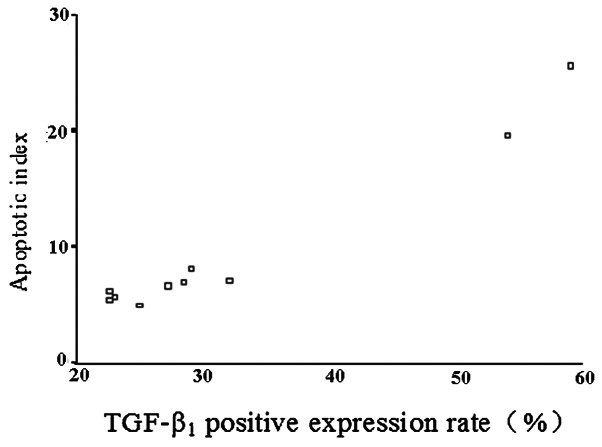
Correlation between the apoptotic index and transforming growth facor (TGF)-β1 positive expression rate.

A large number of apoptotic cells were observed after 8 and 12 h in the CS group. The majority of the apoptotic cells were located at the top of the epidermis of the mucous membrane and a few apoptotic cells were located in the crypt. After counting the number of apoptotic cells in the CN, CS and DCS groups at each time point, the apoptotic index (AI) was calculated (Table III). Correlation analysis of the positive expression rate of TGF-β1 and the AI of the epithelial mucosa of the rat ileal mucous membrane was performed. The results showed that the AI of the epithelial mucosa of rat ileal mucous membrane in each time point in DCS group was lower than the CS group, but still higher than the CN group. The positive expression rate of TGF-β1 in the ileal mucous membrane of the rat epithelial mucosa was positively correlated in the CS group and the DCS group (r=0.980, P<0.05; Fig. 1).

## Discussion

The cold stress reaction is a tension state of the body from the normal state when stimulated by a cold environment. The mechanism by which cold stimulation induces the gastrointestinal stress response is complex and results in gastrointestinal function and mucosal structural changes affecting the nerves and body fluids, as well as other factors. Akimoto *et al*(12) performed a study on the correlation between gastrointestinal mucosal injury and gastrointestinal microcirculation and identified that the gastrointestinal stress response induced by cold stimulation is essentially the process of ischemia-reperfusion of gastrointestinal tissue. Another study demonstrated that the occurrence of apoptosis in the mucosa of the small intestinal leads to the abnormal expression of apoptosis-related genes and further increases the incidence of apoptosis (13).

TGF-β1 promotes apoptosis. In liver cells, TGF-β1 induces apoptosis by activating p38 MAPK (14). TGF-β1 may promote the apoptosis of the CD34 and CD34-DR cells of patients with chronic myeloid leukemia; however, it does not increase the expression of the CD95 Fas receptor in leukemia cells. This condition suggests that TGF-β1 promotes the apoptosis of basal cells via a non-Fas-dependent pathway (15). TGF-β1 promotes the apoptosis of progenitor cells of normal and malignant B lymphocytes; however, it does not change the expression of B cell lymphoma 2 (Bcl-2) (16). The mechanism by which TGF-β1 promotes the apoptosis of small intestinal cells remains unclear.

In the current study, the immunohistochemical detection of TGF-β1, as well as TUNEL and DNA agarose gel protein electrophoresis for the detection of apoptosis, were performed on rats at 2, 4, 8, 12 and 24 h after the establishment of the CS model. The expression levels of TGF-β1 and apoptotic indices of the ileal mucosa epithelial cells in the rats were dynamically detected. The pathological changes of the mucosal epithelial tissue of the rat terminal ileum were observed in the cold-restraint stress state. After 8–12 h of cold-restraint stress, the villi of the small intestine had a disordered arrangement; however, the mucous membrane was complete and not necrotic. After 24 h under cold-restraint stress, the villi were repaired and arranged regularly and edema disappeared. The classic theory states that apoptosis and necrosis are two mutually exclusive methods of cell death. A previous study (17) demonstrated that the discrimination between the two is not absolute; they also often exhibit interrelated phenomena. The results from the DNA agarose gel protein electrophoresis revealed the characteristic ‘ladder’ bands of apoptosis after 8 and 12 h of cold-restraint stress. At each time point, irregular film-like bands that represent cell necrosis were not present. This result suggests that the pathological changes of the epithelial cells of the rat small intestine in the cold-restraint stress state were mainly apoptosis, not necrosis. In this study, TUNEL technology was used to detect the occurrence and distribution of epithelial cell apoptosis. The results revealed that the incidence of apoptosis of the small intestine epithelial cells in the cold-restraint stress state at each time point was significantly higher than that of the NC group. Apoptosis exists at each time point and peaks after 8-12 h of cold-restraint stress. In this study, the apoptotic cells were mainly located at the top of the villi of the small intestine, which corresponds with the results from a previous study (18). However, there is also a alternative viewpoint that apoptosis of small intestine epithelial cells mainly occurs in the crypt of the intestinal glands and only a small amount of apoptotic cells are located on the surface of the intestinal villi, indicating that the apoptosis of cells of the crypt migrate upward (19).

In the current study, the expression of TGF-β1 was detected by immunohistochemistry. We identified that TGF-β1 is expressed in normal rat small intestine epithelial cells. After 2 h of cold-restraint stress, the positive expression rate of TGF-β1 was slightly higher than that of the NC group, and tended to increase with time. After 8 h, it reached a peak. In this study, the experimental results revealed that the AI of the epithelial cells of the rat small intestine is correlated with the positive expression rate of TGF-β1. Continual slices of rat small intestine epithelial tissue were observed. No significant differences were identified in the positive expression rate of TGF-β1 at 2, 4 and 24 h after cold-restraint stress by immunohistochemical staining or in the apoptosis rate compared with those of the NC group. Whereas, after 8 and 12 h of cold-restraint stress, a significant difference was observed compared with that of the NC group. Following treatment with the proteoglycan, desmin, which specifically inhibits the activation of TGF-β1, the rats were subjected to cold-restraint stimulation. Following TUNEL and immunohistochemical staining, the results revealed that the morphology of the rat small intestine epithelium was not significantly different compared with that of the CS group following the specific inhibition of TGF-β activity. However, the positive expression rate of TGF-β1 and AI in the epithelial cells was significantly decreased. The results of the correlation analysis revealed that the apoptosis rate of the rat small intestine epithelial cells is positively correlated with the positive expression rate of TGF-β1. The results revealed that the AI of the rat small intestine epithelial cells in the cold-restraint stress state changes with the positive expression rate of TGF-β1 in the epithelial tissue. TGF-β1 may induce the apoptosis of rat small intestine epithelial cells. Our data also revealed that the positive expression rate of TGF-β1 and the AI were not significantly different after 2, 4 and 24 h of cold-restraint stress from those of the control group. This result may be due to the reduced expression of TGF-β1 in the cell stress reaction at early and post-peak stages in the expression of TGF-β1.

When TGF-β1 is secreted into the blood, the epithelial cells of the small intestine cause apoptosis through the paracrine mechanism, when the intracellular TGF-β1 levels increase. The autocrine mechanism in cells is also able to induce apoptosis. In this study, after injection with TGF-β1 inhibitor decorin, the AI of the rat small intestine epithelial cells decreased along with the positive expression level of TGF-β1. However, this was still higher than that of the control rats in a normal state. This result indicates that other mechanisms are able to induce and promote the apoptosis of epithelial cells in the rat small intestine. However, the specific mechanism of the TGF-β1-induced apoptosis of the rat small intestine epithelial cells remains unclear. Studies have shown that TGF-β1-induced apoptosis in the small intestine is correlated with a high expression level of c-MYC and low expression level of Bcl-2 (20). TGF-β1 causes G1 arrest through a variety of mechanisms (21), including preventing pRB phosphorylation, inhibiting the protein expression of the G1 phase of the cell cycle, inhibiting cyclin-dependent kinase (CDK) activity and increasing CDK levels (22).

In summary, TGF-β1 inhibits cells from passing the G1/S checkpoint through a variety of mechanisms, which arrests cells in the G1 phase. However, the specific mechanism of the TGF-β1-induced apoptosis of the epithelial cells of the rat small intestine has not been investigated in this study due to limited time and conditions, and requires further investigation.

The experimental results revealed that the expression of TGF-β1 was increased in the rat small intestine epithelial cells under cold-restraint stress and apoptosis increased. TGF-β1 may be one of the cytokines that induces the apoptosis of the epithelial cells of the mucous membrane in the small intestine.

## Figures and Tables

**Table I:** Expression of TGF-β1 in the epithelial tissue of rat ileal mucous tissue in the CS group.

Group	Positive expression rate (%)	χ^2^	P-value
Control	16.67		
2 h	22.72	0.024	0.578
4 h	29.17	0.444	0.370
8 h	59.09	7.053	0.009^a^
12 h	54.16	5.689	0.018^a^
24 h	27.27	0.262	0.434

a P<0.05. TGF, transforming growth factor; CS, cold-restraint stress.

**Table II:** Expression of TGF-β1 in the epithelial tissue of rat ileal mucous tissue in the DCS group.

Group	Positive expression rate (%)	χ^2^	P-value
Control	16.67		
2 h	25.00	0.108	0.511
4 h	23.08	0.037	0.560
8 h	32.14	0.840	0.275
12 h	28.57	0.413	0.376
24 h	22.72	0.024	0.578

TGF, transforming growth factor; DCS, cold-restraint stress with drug intervention.

**Table III:** Apoptotic index (AI) of the epithelial mucosa of the rat ileal mucous membrane at each time point.

Group	2 h	4 h	8 h	12 h	24 h
CN	4.41±1.87	4.42±1.86	4.41±1.83	4.43±1.88	4.41±1.87
CS	5.47±1.36	8.14±3.32^a^	25.69±8.09^b^	19.65±6.61^a^	6.65±2.95
DCS	4.92±2.36	5.68±2.59	7.13±2.70^a^	6.91±2.13^a^	6.25±2.71

Data are presented as mean ± standard deviation.

a P<0.05,

b P<0.01. CN, control; CS, cold-restraint; DCS, cold-restraint with drug intervention.
